# The Role of Pseudomonas in Heterotrophic Nitrification: A Case Study on Shrimp Ponds (*Litopenaeus vannamei*) in Soc Trang Province

**DOI:** 10.3390/microorganisms7060155

**Published:** 2019-05-29

**Authors:** Thanh Trung Tran, Nathan J. Bott, Nhan Dai Lam, Nam Trung Nguyen, Oanh Hoang Thi Dang, Duc Hoang Le, Lam Tung Le, Ha Hoang Chu

**Affiliations:** 1 Centre for Environmental Sustainability and Remediation, School of Science, Royal Melbourne Institute of Technology University, PO Box 71, Bundoora Victoria 3083, Australia; trantrungthanh3t@gmail.com (T.T.T.); nathan.bott@rmit.edu.au (N.J.B.); 2 National Key Laboratory of Gene Technology, Institute of Biotechnology, Vietnam Academy of Science and Technology, 18 Hoang Quoc Viet, Cau Giay, Hanoi 100000, Vietnam; nhan@ibt.ac.vn (N.D.L.); nam@ibt.ac.vn (N.T.N.); lehoangduc8x@gmail.com (D.H.L.); letunglam1991@gmail.com (L.T.L.); 3 Faculty of Biotechnology, Graduate University of Science and Technology, Vietnam Academy of Science and Technology, 18 Hoang Quoc Viet, Cau Giay, Hanoi 100000, Vietnam; 4 Department of Aquatic Pathology, College of Aquaculture and Fisheries, Can Tho University, Can Tho 900000, Vietnam; dthoanh@ctu.edu.vn

**Keywords:** *Pseudomonas*, *Litopenaeus vannamei*, heterotrophic nitrification, ammonia

## Abstract

Based on a total of 6,295,650 sequences from the V3 and V4 regions (16S ribosomal RNA), the composition of the microorganism communities in the water of three *Litopenaeus vannamei* (Decapoda, Whiteleg shrimp; Soc Trang, Vietnam) ponds were identified. *Pseudomonas* (10–20.29%), *Methylophilus* (13.26–24.28%), and *Flavobacterium* (2.6–19.29%) were the most abundant genera. The total ammonia (TAN) concentration (*p* = 0.025) and temperature (*p* = 0.015) were significantly correlated with the relative abundance of *Pseudomonas* in two bacterial communities (ST1, ST4), whereas the predictive functions of microorganism communities based on 16S rRNA gene data was estimated using Phylogenetic Investigation of Communities by Reconstruction of Unobserved States (PICRUST), which showed that nitrogen metabolism was significantly negatively correlated (*p* = 0.049) with TAN concentration. The abundance of *Pseudomonas* and nitrogen metabolism increased with a decrease in TAN concentration. The correlation between TAN concentration and the abundance of *Pseudomonas* was followed by the isolation, and heterotrophic nitrifying performance analysis was used to confirm our findings. Six *Pseudomonas* strains capable of heterotrophic nitrification were isolated from the three water samples and showed a complete reduction of 100 mg/L NH_4_Cl during a 96-h cultivation. These results indicate the potential of applying *Pseudomonas* in shrimp ponds for water treatment.

## 1. Introduction

Brackish-water aquaculture is a major worldwide source of food production and an economic driver [1]. Crustacean aquaculture comprises 57% of the world brackish water culture, in which marine decapod shrimps, primarily comprising *Penaeus monodon* and *Litopenaeus vannamei*, accounted for more than 99% of total production in 2010 [2].

In aquaculture systems, ammonia accumulates rapidly due to natural excretion and high metabolic excretion rate in intensive shrimp ponds during the cultivation life-cycle [3,4]. The concentration of total ammonia nitrogen (TAN) at high levels is toxic to shrimp aquaculture, resulting in post-larvae reduced growth under NH_3_ concentrations of 0.301 mg/L [4]. Deterioration of environmental conditions has led to significant economic losses [5]. Changes in environmental parameters have been shown to change the structure of bacterial communities in the water of aquaculture systems [6,7,8,9], and bacteria have been found to play roles in remediating polluted and contaminated water [10] and are correlated with water quality [11]. Information is lacking regarding environmental effects on the relative abundance of bacteria for the monitoring of water quality in shrimp ponds.

High water quality in shrimp ponds is seen as a key requirement for the success and development of shrimp aquaculture [11,12]. Treatments with an intensive microbial community can improve water quality in a pond [13]. *Pseudomonas* has been reported to be a dominant microorganism in aquaculture systems [11], as seen in milkfish (*Chanos chanos*) farming in the Philippines [14], and has been shown to play a role in heterotrophic nitrification activity [15]. However, to date, this is poorly understood [9].

Traditional molecular methods used to discover the abundance and composition of bacterial communities have provided considerable amounts of data on the long-term distribution of dominant bacterial groups in communities [16,17,18,19,20]. However, primary limitations of these methods, including isolation methods and polymerase chain reaction–denaturing gradient gel electrophoresis (PCR-DDGE), are that they are time-consuming and poorly represent the diversity of less common bacterial groups [21]. High-throughput next-generation sequencing (NGS) systems have benefited the study of microbial populations. NGS techniques can provide information about all members in complex communities [22], and have been applied in numerous studies [8,9,13,21].

In this study, the abundance and composition of bacterial communities in the water of *L. vannamei* ponds were investigated by analyzing the V3 and V4 regions of 16S rRNA gene using the Nextera XT index kit (Illumina, San Diego, CA, USA) and sequenced using Miseq reagent kit V3 (600 cycles) on the Illumina MiSeq platform (Illumina, San Diego, CA, USA). To understand the influence of the relative abundance of *Pseudomonas* on environmental factors, Pearson correlation analyses were performed. The relationship between environmental factors and predictive functions of bacterial communities were analyzed to confirm the dynamic change in bacterial abundance under different environmental conditions. Finally, the activity of *Pseudomonas* isolates for ammonia reduction was examined.

## 2. Materials and Methods

### 2.1. Samples Collection

Water samples were collected three times every 2 weeks at four geographic positioning system (GPS) locations (Table S1) at Lich Hoi Thuong town, Tran De district, Soc Trang province, which is a large shrimp farming field in Vietnam, during November 2015 before the harvest period. *L. vannamei* ponds (Soc Trang 1, Soc Trang 3, and Soc Trang 4) were sampled in triplicate and one *P. monodon* pond (Soc Trang-*Penaeus monodon*) as a control sample for comparison. The four selected shrimp ponds are semi-intensive, do not use probiotic products, and have the same input water source. The start of cultivation of these four shrimp ponds was the same. Physicochemical characteristics were measured three times during the sampling period, as shown in Table S1. At each of the four GPS locations, water samples were collected according to the protocol described by Tang et al. [8]. Water samples were collected at five different sites in each pond using a 10 L sterile plastic bottle and stored in dry ice. Bacterial cells were separated using a standard sequential filtration technique: Each water sample was filtered through 8-µm qualitative filter paper to remove large suspended participles, and 1.0 L filtrate was subsequently filtered through polycarbonate membranes with a 0.8 to 0.22-µm pore size (47 mm diameter, Whatman^®^, Loughborough, England). Total genomic DNA was extracted in triplicate from three 0.8 to 0.22-μm membranes of each sample using the QIAamp^®^ DNA Stool Mini Kit (Qiagen, Germantown, MD, USA) following the manufacturer’s instructions. Total extracted DNA mixtures containing an equivalent amount of DNA from pooled samples were used as template for PCR sequencing using the Nextera XT index kit (Illumina, San Diego, CA, USA) and sequenced by Miseq reagent kit V3 (600 cycles) (Illumina, San Diego, CA, USA) to generate paired-end reads at Macrogen, Seoul, South Korea on the Illumina MiSeq platform (Illumina, San Diego, CA, USA).

### 2.2. Sequence Processing and Analysis

At Macrogen (Seoul, South of Korea), bacterial 16S rRNA gene amplicons were sequenced on the Illumina MiSeq platform (Illumina, San Diego, CA, United States) (2 × 250 bp paired-end reads) and demultiplexed to remove all indexes, primers, and barcodes. Low quality (≤25) and ambiguous bases were removed using Trimmomatic version 0.38. All quality reads were then analyzed using the Greenfield Hybrid Analysis Pipeline (GHAP) (Commonwealth Scientific and Industrial Research Organisation (CSIRO), Australia). A minimum overlap of 20 bp was applied to join forward (R1) and reversed (R2) reads. Minimum and maximum lengths of 260 bp and 480 bp, respectively, were selected for classification analysis using the GHAP pipeline. This pipeline was based on the Usearch tools and the Ribosomal Database Project (RDP) classifier. The Green genes reference database was used to assign taxonomy to the Operational Taxonomic unit (OTU). Following this, macQIIME (version 1.9.1-20150604, Werner lab) was used to analyze the alpha diversity (ACE, chao1, Shannon–Wiener index (H), Simpson index (D)) and beta diversity. Alpha diversity was used to calculate the within-community diversity for each of the four microorganism communities. A sequence depth of 20,000 reads per samples was selected to analyze the alpha diversity metrics with 1000 iterations. To compare samples, beta diversity was computed from the phylogenetic tree with both weighted and unweighted UniFrac distances under the sequence depth threshold of 20,000 reads using the jackknifed_beta_diversity.py command.

### 2.3. Predictive Metagenome Functions Analysis

Phylogenetic Investigation of Communities by Reconstruction of Unobserved States (PICRUST) software package [23] was used to predict the metagenome functional of all bacterial communities using 16S rRNA gene sequencing based on the Kyoto Encyclopedia of Genes and Genomes (KEGG) database. The pick_reference_otus_through_otu_table.py function was used to pick up OTUs assigned to Green genes database version 4 February 2011, at 97% identity to match the database with PICRUST before analysis. Online Galaxy version 1.0.0 of PICRUST was used for these three steps. Normalize by copy number step was analyzed to correct the OTUs table of all samples. Based on KEGG orthologs, a metagenome prediction step was performed. Three levels of KEGG pathways were matched using a categorized by function step.

### 2.4. Statistical Analysis

Welch’s *t*-test was used to compare the alpha diversity metrics between samples. Pairwise correlations between two factors among relative abundance of *Pseudomonas*, environmental parameters, and predictive metagenomics functions were analyzed using the Pearson correlation formula with “ggscatter” function of the “ggpubr” package in Comprehensive R Archive Network (CRAN) based on RStudio (Boston, MA, USA). Based on three replicates of each index from two water samples, the statistical analyses were calculated.

### 2.5. Isolation and Identification of Heterotrophic Nitrifying Bacteria

We added 100 mL water samples from the three *L. vannamei* ponds to an enrichment medium (Peptone 20 g, glycerol 10 mL, K_2_HPO_4_ 1.5 g, MgSO_4_·7H_2_O 1.5 g, NaCl 20 g, and Triclosan 0.02) and incubated for 24 h at 30 °C with shaking at 200 rpm to enhance *Pseudomonas* growth. Enriched solutions were diluted serially and plated on meat peptone agar (MPA; beef extract 5 g/L, peptone 10 g/L, NaCl 15 g/L, agar 15 g/L, pH 7 ± 0.2) and on King B medium and incubated at 30 °C for 24 h with shaking at 200 rpm. After incubation, counts were calculated by the total colonies on MPA and fluorescent colonies on King B medium under ultraviolet (UV) light at 365 nm. Colonies with potential ammonia-oxidizing activities were then isolated by cultivation on King B medium with the addition of 100 mg/L NH_4_Cl. Following this, colonies were confirmed by morphological tests (mobility and staining), biochemical tests (catalase test, carbohydrate test, oxidase test, and gelatin liquefaction), and 16S rRNA gene sequences. 16S sequences were amplified using universal PCR primers: 27F (AGAGTTTGATCCTGGCTCAG) and 1492R (GGTTACCTTGTTACGACTT), sequenced at Macrogen Inc. (Seoul, Korea) and classified using BLAST on NCBI. A phylogenetic tree was constructed using the neighbor-joining method in Molecular Evolutionary Genetics Analysis (MEGA) version 7.0.

### 2.6. Ammonia-Oxidizing Activity Test

Isolated potential ammonia-oxidizing colonies were cultivated on the heterotrophic nitrification medium (HNM) (K_2_HPO_4_ 7.0 g/L, KH_2_PO_4_ 3.0 g/L, MgSO_4_·7H_2_O 0.1 g/L, NH_4_Cl 1.0 g/L, FeSO_4_·7H_2_O 0.05 g/L, and CH_3_COONa 10 g/L; pH 7.2) [24] at 30 °C for 24 h before testing. The ammonia-oxidizing activity of *Pseudomonas* was determined using phenate methods.

## 3. Results

### 3.1. Alpha Diversity and Beta Diversity Analysis

The analysis of 16S rRNA gene sequences in the V3 to V4 regions from our samples (Table 1) showed that we obtained a total of 6,295,650 high-quality sequences, 99.99% of which were matched as bacteria, while 0.006% were matched as archaea. The remaining 0.004% of sequences were unclassified. The cut-off threshold of 3% (97% similarity) applied in this study is a common rule for operational taxonomic units (OTUs) clusters [25]. Welch’s *t*-test (α = 0.05) results showed alpha diversity metrics separated differently among samples. In particular, all *p*-values (Table S2) from Welch’s *t*-test analysis between samples were higher than 0.05. The highest bacterial richness values, represented by the number of observed OTUs in samples, were observed in Soc Trang–*Penaeus monodon* (ST-PM) sample and lowest were observed in the ST3 sample. The richness estimator containing abundance-based coverage estimator (ACE) of species richness and Chao richness estimator (Chao1) [26] had similar trends. The highest ACE value of 1861.68 was found in ST-PM and the lowest, 1343.40, in ST3. The values of ACE and Chao1 were all higher than observed OTUs, indicating that a higher number of species would be expected when the number of observed OTUs increases. The diversity estimation, including the Shannon diversity index (Shannon) and the Simpson diversity index (Simpson) [27], revealed the difference from the richness estimator values. The highest Shannon and Simpson values were in ST1 (5.54 and 0.95, respectively), whereas the Shannon and Simpson values in ST3 were the lowest, at 4.47 and 0.86, respectively. That means ST3 was the most diverse. Rarefaction curves from all samples (Figure S1) did not reach saturation, which indicated more observed OTUs would be expected to be found in all samples.

### 3.2. Compositions and Abundances of Bacterial Communities Analysis

**Figure 1:**
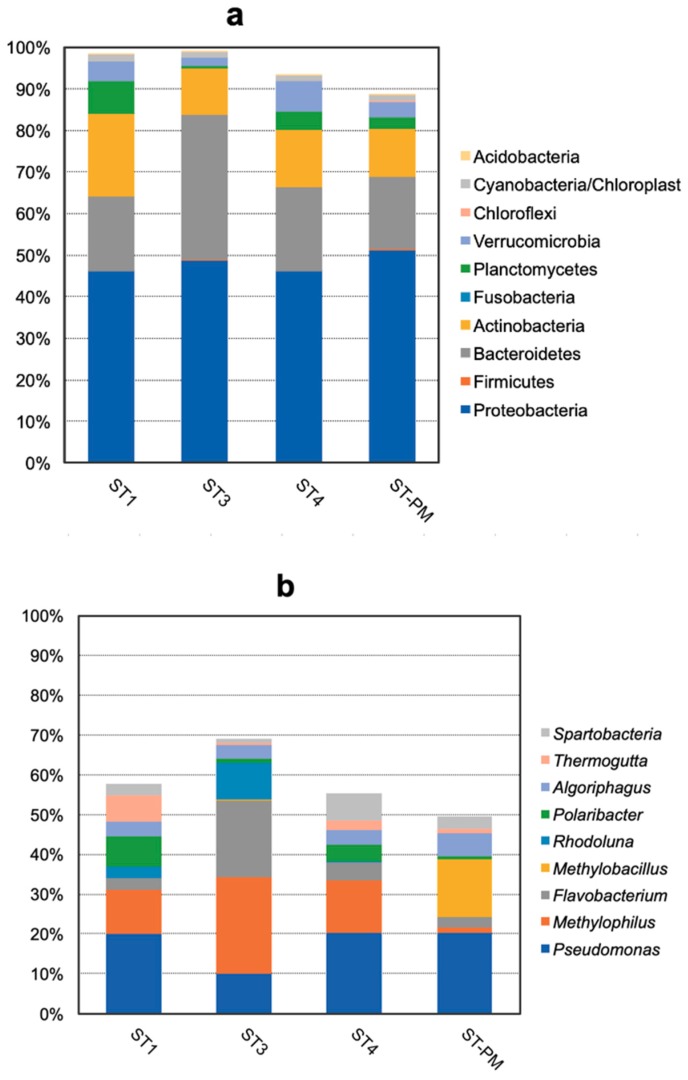
Distribution of the top 10 most common (**a**) phyla and (**b**) genus. ST1, ST3, ST4: water samples *Litopenaeus vannamei* from three different ponds; ST-PM: a water sample of *Penaeus monodon*.

The top 10 phyla with highest numbers of assigned sequences accounted for over 90% of the total sequences from water samples, as shown in Figure 1a. This shows that Proteobacteria was the dominant phylum, ranging from 46.2% to 48.6% of the total bacterial population. The second most dominant phylum was Bacteroidetes (18.1–34.8%), followed by Actinobacteria (11.3–19.8%) in the three *L. vannamei* ponds. This result indicates that the distribution of the top 10 microorganism phyla in the three *L. vannamei* communities was similar in comparison to the distribution of the top 10 phyla in the *Penaeus monodon* community.

The top 10 genera, shown in Figure 1b, with higher numbers of assigned sequences, accounted for almost 50% of the total sequences in the water samples. The result shows that *Pseudomonas* was the dominant genus (10.03–20.29%) in the water samples other than in ST3 (10.03%) where *Pseudomonas* had a lower number of assigned sequences than *Methylophilus* (13.26–24.28%) and *Flavobacterium* (2.6–19.29%). This result indicates that the distributions of the top 10 microorganism genera among the three *L. vannamei* ponds were similar in comparison with the *P. monodon* pond (ST-PM). The second most dominant genus in ST-PM was *Methylobacillus*. 

### 3.3. Predictive Functions of Microorganism Communities Analysis by PICRUST

**Figure 2:**
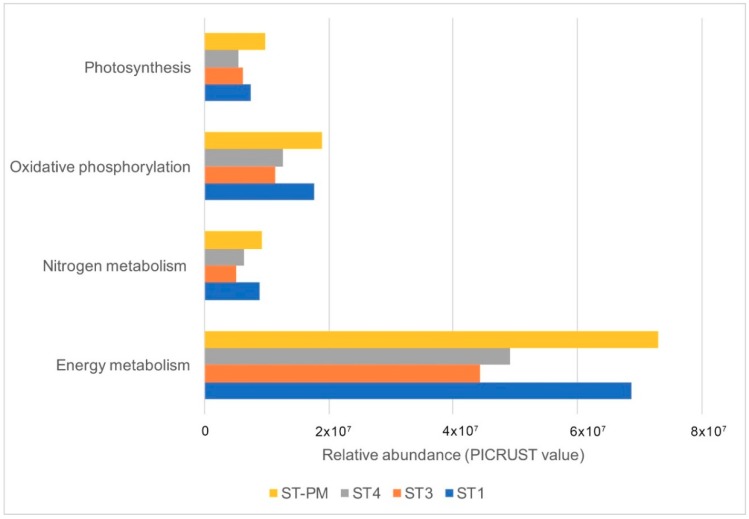
The relative abundance of predictive metagenome functions from samples. ST1, ST3, ST4: three water samples of *L. vannamei;* ST-PM: a water sample of *P. monodon.*

The differences in the predictive functions of microorganism communities based on 16S rRNA gene data using Phylogenetic Investigation of Communities by Reconstruction of Unobserved States (PICRUST) software are shown in Figure 2. Figure 2 shows the KEGG ortholog (KO) abundance assigned to energy metabolism, which accounted for 20.68% of the total copy number (PICRUST value). In this analysis, we focused on the abundance of KEGG orthologs assigned to nitrogen metabolism (5.3%), oxidative phosphorylation (2.6%), and photosynthesis (2.6%), which represent energy metabolism. This showed that the numbers of copies (all pathways) assigned to energy metabolism from ST1 (68,645,845) and ST-PM (72,878,280) were higher than ST3 (44,246,846) and ST4 (49,132,370). For nitrogen metabolism, oxidative phosphorylation, and photosynthesis, the OTU abundance fluctuated minimally, but the trends were similar. 

### 3.4. Relationship Among Environmental Factors, Predictive Functions, and Bacterial Abundance

Comparison of the value of the predictive functions from different communities showed significant changes in the influence of TAN concentration on nitrogen metabolism (*r* = −0.95, *p* = 0.049). The observed negative correlations between TAN concentration and the three processes, oxidative phosphorylation (*r* = −0.94, *p* = 0.065), photosynthesis (*r* = −0.81, *p* = 0.19), and energy metabolism (*r* = −0.94, *p* = 0.063), were not significant. The considerable decrease in TAN concentration was correlated with the increase in nitrogen metabolism.

The insignificant correlations between the abundance of bacteria in shrimp ponds and environmental parameters including biochemical oxygen demand (BOD) (*r* = −0.0045, *p* = 1), Chlorophyll-a (*r* = 0.4, *p* = 0.6), chemical oxygen demand (COD) (*r* = −0.47, *p* = 0.53), NO_3_-N (*r* = 0.37, *p* = 0.63), pH (*r* = −0.68, *p* = 0.32), PO_4_-P (*r* = −0.35, *p* = 0.65), total ammonia nitrogen (TAN) (*r* = −0.87, *p* = 0.13), total dissolved solids (TDS) (*r* = 0.56, *p* = 0.44), total organic matter (TOM) (*r* = −0.66, *p* = 0.34), total N (*r* = −0.34, *p* = 0.66), total P (*r* = −0.29, *p* = 0.71), total suspended solids (TSS) (*r* = −0.65, *p* = 0.35) are shown in Figure S4. The abundance of bacteria was significantly positively related to temperature (*r* = 0.96, *p* = 0.036).

The results of pairwise correlation analysis showed that Total Ammonia (TAN; *r* = −0.97, *p* = 0.025) and temperature (*r* = 0.99, *p* = 0.015) were significantly correlated with the relative abundance of *Pseudomonas* (Figure S5). The relative abundance of *Pseudomonas* decreased with the increase in TAN concentration. The correlations between the relative abundance of *Pseudomonas* and the 11 environmental parameters were not significant (Table 2).

### 3.5. Isolation, Identification, and Heterotrophic Nitrifying Activity

**Figure 3:**
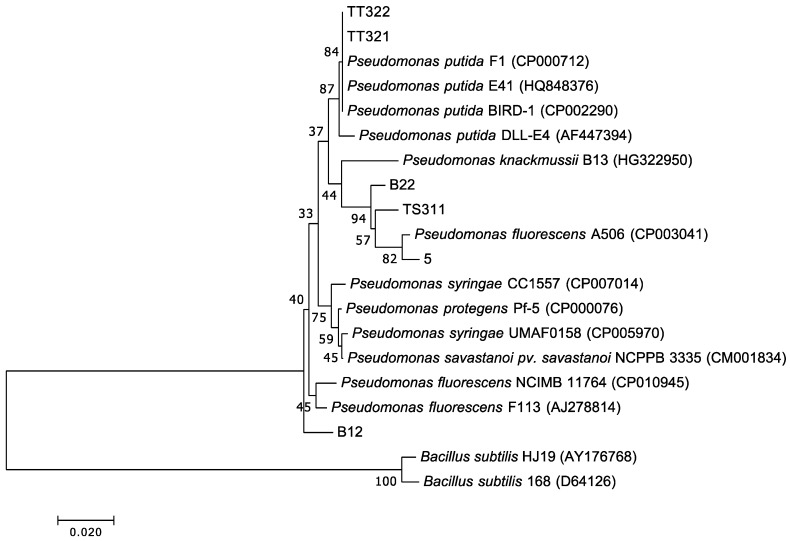
Neighbor-joining tree base on 16S ribosomal RNA sequences reveals the phylogenetic position of six isolates and representatives of several other reference sequences. Six strains (B12, B22, 5, TS311, TT321, and TT322) were isolated from three water samples of *L. vannamei* are *Pseudomonas* sp.

The number of total aerobic bacteria (colony forming units (CFU)/mL) and *Pseudomonas* (CFU/mL) in Table S3 show that *Pseudomonas* were efficiently enriched. Twelve potential ammonia-oxidizing colonies were isolated on King B medium with the additional of 100 mg/L NH_4_Cl (Table S4). After confirmation experiments (Table S5 and Figure S6), six isolates were found. The sequences of the 16S rRNA gene from six isolated colonies are provided in Table S6 and classified into *Pseudomonas* sp. The morphological and biochemical characteristics and phylogenetic tree analysis (Figure 3) of six strains indicated that all strains are *Pseudomonas* sp.

**Figure 4:**
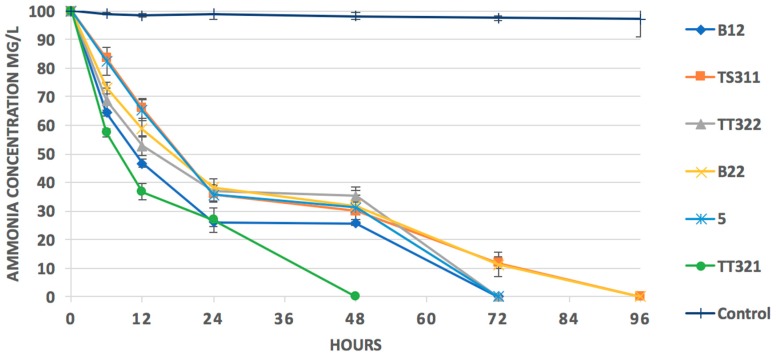
Changes in concentrations of N-NH_4_^+^ in the culture medium during the heterotrophic nitrification test. Six strains (B12, B22, 5, TS311, TT321, and TT322) were isolated from three water samples of *L. vannamei*.

The results of the ammonia-oxidizing activity test in Figure 4 indicated that after 96 h cultivation, the ammonia concentration of 100 mg/L had been completely removed by the six colonies. 

## 4. Discussion

In this study, we identified bacterial compositions at phylum and genus levels to understand the diversity and dominance within bacterial communities of three *L. vannamei* ponds. Environmental parameters were evaluated to understand the significant influence on the relative abundance of *Pseudomonas*. The observed correlation between environmental factors and predictive metagenomics function was clarified the dynamic change of metabolism in different conditions of water. This correlation also suggested the role of related bacterial groups. The performance of *Pseudomonas* isolates in regarding the heterotrophic nitrification was examined to confirm the hypothesis about the correlation between the relative abundance of *Pseudomonas* and TAN concentration.

There were apparent differences among the composition of bacterial communities in three *L. vannamei* ponds and a *P. monodon* pond. Our result clearly revealed variation in phylum composition among different communities. Although there are some fluctuations in the percentage in three *L. vannamei* ponds at the phylum level, they shared the similar dominant orders (Figure 1a). The dominant phyla in our study were: Proteobacteria (46.2–48.6%), Bacteroidetes (18.1–34.8%), and Actinobacteria (11.3–19.8%), while Cardona [13] in other research for clear water in shrimp tanks reported that Proteobacteria was the most dominant phylum (50.4–60%), Bacteroidetes was second-most dominant phylum (21.9–30%), and Cyanobacteria was third-most dominant phylum (8.5–13%). Another publication [21] reported that the bacterial compositions at phylum level in surface water were the mostly dominated by Proteobacteria (24.28–48.22%), followed by Cyanobacteria (5.9–37.61%), Bacteroidetes (5.4–34.82%), and Actinobacteria (8.2–22.37%).

At the genus level, the composition of bacteria in shrimp pond water showed differences from the study of Tang and colleagues [8]. Their results indicated that the dominant genera in shrimp pond water were *Mycrobacterium* (1.523%), *Conexibacter* (1.055%), *Hydrogenophaga* (0.922%), and *Rhodobacter* (0.609%), while the dominant genus in our results were *Pseudomonas* (10–20.29%), *Methylophilus* (13.26–24.28%), and *Flavobacterium* (2.6–19.29%). A key difference between the two analyses was the different number of total high-quality sequences, which was 6,295,650 in our study in comparison with 18,147 in the study from Tang and colleagues. Our results are consistent with a report on the population of Pseudomonadaceae, which has been frequently considered as dominant in aquaculture systems [11,14]. In this study, the second most dominant genus in a *P. monodon* pond was *Methylobacillus* (14.30%) which was different from *L. vannamei* ponds with *Methylophilus*. The dominance of *Methylophilus* in ST3 was a novel discovery in bacterial research in *L. vannamei* pond water.

Our study, based on our limited sampling, indicates that environmental parameters significantly influence the composition of bacterial communities. This was consistent with previous studies [28,29,30]. Fortnightly water sampling provided a snapshot of water chemistry characteristics in shrimp ponds before the harvest period. In our study, water temperature had a significant positive influence on the relative abundance of bacteria and *Pseudomonas* in particular. This result was consistent with previous analyses [6,8,11,21], in that the increase of bacterial abundance significantly correlates with higher water temperature. Our results also indicated that TAN concentration was significantly negatively correlated with *Pseudomonas* population, while insignificant correlations between the relative abundance of *Pseudomonas* with all other environmental parameters were shown.

We hypothesized that there may be considerable differences among the populations of *Pseudomonas* based on differing amounts of TAN concentrations in these shrimp ponds. However, further studies are needed to confirm this hypothesis for investigating which specific species or the specific *Pseudomonas* group play the heterotrophic nitrification role and the correlation between these groups and environmental parameters. The difference in genus composition may reflect the dynamics of environmental parameters. This was demonstrated based on the results of this study with the significant correlation between *Pseudomonas* and two environment parameters, TAN and temperature. TAN concentration was dramatically decreased when the population of *Pseudomonas* was rising. A strong significant correlation suggested that *Pseudomonas* plays an important role in maintaining water quality in shrimp ponds due to their heterotrophic nitrification activity. 

We found a significant negative correlation (*r* = −0.95, *p* = 0.049) between the TAN concentration and nitrogen metabolism, and an insignificant relationship (*r* = 0.84, *p* = 0.16) between temperature and nitrogen metabolism. This is consistent with a previous study that showed that bacterial communities in the water of shrimp ponds undergo a change in energy metabolism under long-term ammonia toxicity [4]. This hypothesis was confirmed by the isolation and heterotrophic nitrifying performance of *Pseudomonas* strains. After the enrichment, the morphological and biochemical characteristics and phylogenetic tree analysis enabled the isolation of six *Pseudomonas* strains. The six colonies showed their ammonia-oxidizing activities after 96 h of cultivation. This is consistent with previous studies [31,32,33,34], indicating that *Pseudomonas* has the potential for application in ammonium removal treatments.

## 5. Conclusions

In conclusion, the presence of *Pseudomonas* may be considered an important factor in water quality monitoring with regard to ammonia oxidization. The heterotrophic nitrification by *Pseudomonas* suggests that the use of probiotic products to boost the growth of *Pseudomonas* population and optimizing the conditions to maintain sustainable growth of *Pseudomonas* would have some benefit to environmental health. Knowledge about the mechanisms employed by *Pseudomonas* to reduce the TAN concentration and what species within *Pseudomonas* play a key role in heterotrophic nitrification activity are areas that require further examination.

## Figures and Tables

**Table 1:** Alpha diversity analysis based on 16S rRNA gene data from samples.

Sample	Ace	Chao1	Observed OTUs	Shannon	Simpson
ST3	1343.40	1358.76	1049.00	4.47	0.86
ST1	1644.29	1664.06	1341.00	5.54	0.95
ST4	1778.10	1772.69	1363.00	5.05	0.92
ST-PM	1861.68	1848.19	1426.00	5.04	0.93

Principal component analysis (PCA) based on weighted and unweighted UniFrac distances in beta diversity analysis (Figures S2 and S3) showed that the bacterial communities were separate among ST1, ST3, and ST4.

**Table 2:** Pearson correlation between the abundance of *Pseudomonas* and environmental parameters. *r* indicates Pearson correlation.

Parameters	Pearson Correlation Formula	
	*r*	*p*
pH	−0.88	0.12
Temperature	0.99	0.015
BOD	−0.17	0.83
COD	−0.69	0.31
Chlorophyll-a	0.19	0.81
TOM	−0.67	0.33
Total N	−0.6	0.4
Total P	−0.53	0.47
NO_3_-N	0.097	0.9
PO_4_-P	−0.58	0.42
TAN	−0.97	0.025
TDS	0.59	0.41
TSS	−0.86	0.14

## Supplementary Materials

The following are available online at https://www.mdpi.com/2076-2607/7/6/155/s1, Figure S1: title, Table S1: title, Video S1: title. Data Availability: [ST1]: [ Sample name: WWL1], [BioSample: SAMN06062063], [NCBI]; [ST3], [Sample name: WWL3], [BioSample: SAMN06062065], [NCBI]; [ST4], [Sample name: WWL4], [BioSample: SAMN06062066], [NCBI]; [ST-PM], [Sample name: WBT], [BioSample: SAMN06062062], [NCBI].
