# Supplementary material for: The Role of Pseudomonas in Heterotrophic Nitrification: A Case Study on Shrimp Ponds (Litopenaeus vannamei) in Soc Trang Province
Source: Microorganisms. 2019 May 29;7(6):155. doi: 10.3390/microorganisms7060155 (PMC6616971; doi:10.3390/microorganisms7060155)
Supplement: Supplementary file 1 [file microorganisms-07-00155-s001.pdf]

The role of *Pseudomonas* in heterotrophic nitrification: A case study on shrimp ponds (*Litopenaeus vannamei*) in Soc Trang province

Thanh Trung Tran[1,2,3], Nathan J. Bott[1], Nhan Dai Lam[2], Nam Trung Nguyen[2,3], Oanh Hoang Thi Dang[4], Duc Hoang Le[2,3], Lam Tung Le[2], and Ha Hoang Chu[2,3*]

[1] School of Science & Centre for Environmental Sustainability and Remediation, RMIT University, PO Box 71, Bundoora, 3083, Victoria, Australia.

[2] National Key Laboratory of Gene Technology, Institute of Biotechnology, 18 Hoang Quoc Viet, Cau Giay, Ha Noi, Vietnam.

[3] Graduate University of Science and Technology, VAST, 18 Hoang Quoc Viet, Cau Giay, Hanoi, Vietnam

[4]College of Aquaculture and Fisheries, Can Tho University, Can Tho, Vietnam.

[*]Correspondence and requests for materials should be addressed to Assoc. Prof. Ha Hoang Chu (email: chuhoangha@ibt.ac.vn)

Supplementary information

Table S1. The measurement of environmental parameters in shrimp ponds water.

	ST1	ST3	ST4	ST-PM
GPS	9°25’11.3”N 106°08’11.3”E	9°25’06.8”N 106°08’09.6”E	9°25’08.8”N 106°08’05.2”E	9°25’11.7”N 106°08’16.2”E
pH	8.49 ± 0.06	8.74 ± 0.11	8.67 ± 0.06	8.48 ± 0.20
Temperature (°C)	32.90 ± 0.50	32.37 ± 0.81	32.87 ± 0.78	33.00 ± 0.44
BOD (mg l^{-1})	7.10 ± 0.10	11.57 ± 0.67	10.53 ± 0.84	12.23 ± 1.12
COD (mg l^{-1})	22.97 ± 0.60	30.87 ± 0.65	28.57 ± 0.85	26.97 ± 0.50
Chlorophyll-a (µg l^{-1})	107.30 ± 5.83	24.40 ± 0.46	230.67 ± 0.85	30.90 ± 4.87
TOM (%)	2.83 ± 0.35	7.43 ± 0.31	3.48 ± 0.56	5.20 ± 0.10
Total N (mg l^{-1})	1.10 ± 0.09	2.42 ± 0.02	3.34 ± 0.05	0.29 ± 0.06
Total P (mg l^{-1})	0.70 ± 0.05	1.03 ± 0.02	1.46 ± 0.05	0.27 ± 0.03
NO_3-N (mg l^{-1})	0.15 ± 0.01	0.04 ± 0.01	1.03 ± 0.07	0.06 ± 0.01
PO_4-P (mg l^{-1})	0.28 ± 0.04	0.42 ± 0.03	0.56 ± 0.05	0.10 ± 0.01
TAN (mg l^{-1})	0.39 ± 0.01	1.20 ± 0.03	0.69 ± 0.03	0.01 ± 0.01
TDS (mg l^{-1})	143.40 ± 0.70	320.87 ± 1.39	313.67 ± 6.43	2337.17 ± 4.94
TSS (mg l^{-1})	7.07 ± 0.15	14.90 ± 0.30	12.80 ± 0.66	7.80 ± 0.56

Table S2. Welch's t test analysis ($\alpha = 0.05$)

Number	Samples test	P-value
1	ST1 – ST3	0.724770
2	ST1 – ST4	0.927133
3	ST1 – STPM	0.869478
4	ST3 – ST4	0.663515
5	ST3 – STPM	0.615345
6	ST4 – STPM	0.941768

Table S3. Total aerobic bacteria and *Pseudomonas* from samples

Number	Label	Total aerobic bacteria (CFU/ml)	*Pseudomonas*	
			CFU/ml	Isolated strains
1	ST4	$2,5.10^5$	$6,9.10^2$	4
2	ST1	$1,3.10^5$	$3,1.10^2$	1
3	ST3	$3,1.10^4$	$3,6.10^2$	1

Table S4. Evaluate the growth of isolated strains on different medium

Number	Label	HMN medium with NH_4Cl (100 mg/l)
1	TT126	-
2	TT322	+++
3	PS	+
4	B22	+++
5	TS112	++
6	TS311	+
7	TT122	+++
8	5	+++
9	B12	++
10	TT321	+++
11	TS111	-
12	TT121	+++

+++ : Well-gowth

\+ : Grow

\- : None

Table S5. Morphology of isolated strains

	Label	Colour	Shape	Size µm	Gram	Spore-forming
**1**	**PS**	**White**	**Rod**	**0,5x1,5**	**-**	**-**
**2**	**TT322**	**White**	**Rod**	**0,4x1,5**	**-**	**-**
3	TS112	White	Rod	1 x 2,1	+	+
4	B22	White	Rod	0,5x 1,7	+	+
**5**	**TS311**	**White**	**Rod**	**0,5x 1,5**	**-**	**-**
6	TT122	White	Rod	0,8x 1,9	+	+
**7**	**5**	**White**	**Rod**	**0,4x 1,4**	**-**	**-**
**8**	**B12**	**White**	**Rod**	**1 x 2,1**	**-**	**-**
**9**	**TT321**	**White**	**Rod**	**1 x 2,1**	**-**	**-**
10	TT121	White	Rod	0,8x 1,9	+	+

Table S6. 16S rRNA sequences of six *Pseudomonas* isolates

		Sequence
1	B 22	CCTACGGGAGGCAGCAGTGGGGAATATTGGACAATGGGCGAAAGCCTGATCCAGCCATGCCGCGTGTGTGAAGAAGGTCTTCGGATTGTAAAGCACTTTAAGTTGGGAGGAAGGGCAGTAAATTAATACTTTGCTGTTTTGACGTTACCGACAGAATAAGCACCGGCTAACTCTGTGCCAGCAGCCGCGGTAAGACAGAGGGTGCAAGCGTTAATCGGAATTACTGGGCGTAAAGCGCGCGTAGGTGGTTAGTTAAGTTGGATGTGAAGTCCCCGGGCTCAACCTGGGAACTGCATTCAAAACTGACTGACTAGAGTATGGTAGAGGGTGGTGGAATTTCCTGTGTAGCGGTGAAATGCGTAGATATAGGAAGGAACACCAGTGGCGAAGGCGACCACCTGGACTGATACTGACACTGAGGTGCGAAAGCGTGGGGAGCAAACAGGATTAGATACCCTGGTAGTC
2	T T3 22	CCTACGGGAGGCAGCAGTGGGGAATATTGGACAATGGGCGAAAGCCTGATCCAGCCATGCCGCGTGTGTGAAGAAGGTCTTCGGATTGTAAAGCACTTTAAGTTGGGAGGAAGGGTTGTAGATTAATACTCTGCAAGTTTGTCGTTACCGACAGAATAAGCACCGGCTAACTCTGTGCCAGCAGCCGCGGTAATACAGAGGGTGCAAGCGTTAATCGGAATTACTGGGCGTAAAGCGCGCGTAGGTGGTTAGTTAAGTTGGATGTGAAATCCCCGGGCTCAACCTGGGAACTGCATTCAAAACTGACTGACTAGAGTATGGTAGAGGGTGGTGGAATTTCCTGTGTAGCGGTGAAATGCGTAGATATAGGAAGGAACACCAGTGGCGAAGGCGACCACCTGGACTGATACTGACACTGAGGTGCGAAAGCGTGGGGAGCAAACAGGATTAGATACCCTTGTAGTC
3	T S3 11	CCTACGGGAGGCAGCAGTGGGGAATATTGGACAATGGGCGAAAGCCTGATCCAGCCATGCCGCGTGTGTGAAGAAGGTCTTCGGATTGTAAAGCACTTTAAGTTGGGAGGAAGGGCAGTAAATTAATACTTTGCTGTTTTGACGTTACCGACAGAATAAGCACCGGCTAACTCTGTGCCAGCAGCCGCGGTAATTCAGAGGGTGCAAGCGTTAATCGGAATTACTGGGCGTAAAGCGCGCGTAGGTGGTTAGTTAAGTTGGATGTGAAATCCCCGGGCTCAACCTGGGAACTGCATTCAAAACTGACTGACGAGAGTATGGTAGAGGGTGGTGGAATTTCCTGTGTAGCGGTGAAATGCGTAGATATAGGAAGGAACACCAGTGGCGAAGGCGACCACCTGGACTGATACTGACACTGAGGTGCGAAAGCGTGGGGAGCAAACAGGATTAGATACCCGTGTAGTC
4	5	CCTACGGGAGGCAGCAGTGGGGAATATTGGACAATGGGCGAAAGCCTGATCCAGCCATGCCGCGTGTGTGAAGAAGGTCTTCGGATTGTAAAGCACTTTAAGTTGGGAGGAAGGGTTGTAGATTAATACTCTGCAATTTTGACGTTACCGACAGAATAAGCACCGGCTAACTCTGTGCCAGCAGCCGCGGTAATACAGAGGGTGCAAGCGTTAATCGGAATTACTGGGCGTAAAGCGCGCGTAGGTGGTTAGTTAAGTTGGATGTGAAATCCCCGGGCTCAACCTGGGAA

		CTGCATTCAAAACTGACTGACGAGAGTATGGTAGAGGGTGGTGGAATTTCCTGTGTAGCGGTGAAATGCGTAGATATAGGAAGGAACACCAGTGGC<br>GAAGGCGACCACCTGGACTGATACTGACACTGAGGTGCGAAAGCGTGGGGAGCAAACAGGATTAGATACCCTTGTAGTC
5	B 12	CCTACGGGAGGCAGCAGTGGGGAATATTGGACAATGGGCGAAAGCCTGATCCAGCCATGCCGCGTGTGTGAAGAAGGTCTTCGGATTGTAAAGCAC<br>TTTAAGTTGGGAGGAAGGGCATTAACCTAATACGTTAGTGCTTTGACGTTACCGACAGAATAAGCACCGGCTAACTCTGTGCCAGCAGCCGCGGTAA<br>TACAGAGGGTGCAAGCGTTAATCGGAATTACTGGGCGTAAAGCGCGCGTAGGTGGTTTGTTAAGTTGAATGTGAAATCCCCGGGCTCAACCTGGGA<br>ACTGCATCCAAAACTGGCAAGCTAGAGTATGGTAGAGGGTAGTGGAATTTCCTGTGTAGCGGTGAAATGCGTAGATATAGGAAGGAACACCAGTGG<br>CGAAGGCGACTACCTGGACTGATACTGACACTGAGGTGCGAAAGCGTGGGGAGCAAACAGGATTAGATACCCTGGTAGTC
6	T T3 21	CCTACGGGAGGCAGCAGTGGGGAATATTGGACAATGGGCGAAAGCCTGATCCAGCCATGCCGCGTGTGTGAAGAAGGTCTTCGGATTGTAAAGCAC<br>TTTAAGTTGGGAGGAAGGGCAGTAAATTAATACTTTGCTGTTTTGACGTTACCGACAGAATAAGCACCGGCTAACTCTGTGCCAGCAGCCGCGGTAAT<br>ACAGAGGGTGCAAGCGTTAATCGGAATTACTGGGCGTAAAGCGCGCCTAGGTGGTTAGTTAAGTTGGATGTGAAATCCCCGGGCTCAACCTGGGAA<br>CTGCATTCAAAACTGACTGACTAGAGTATGGTAGAGGGTGGTGGAATTTCCTGTGTAGCGGTGAAATGCGTAGATATAGGAAGGAACACCAGTGGC<br>GAAGGCGACCACCTGGACTGATACTGACACTGAGGTGCGAAAGCGTGGGGAGCAAACAGGATTAGATACCCTTGTAGTC

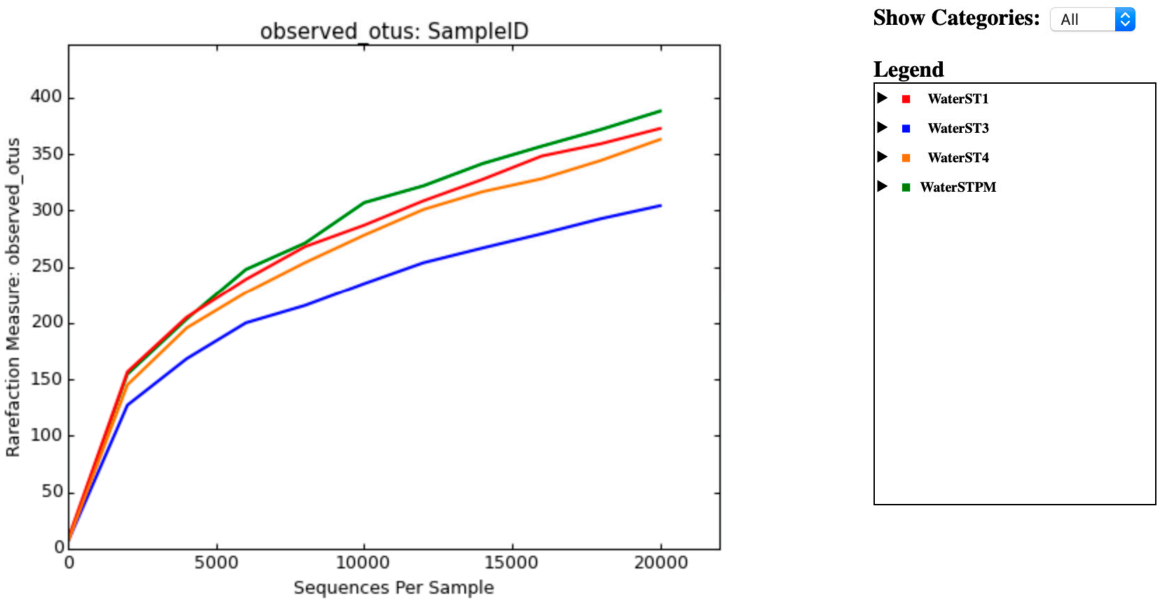

Figure S1. Rarefaction curves

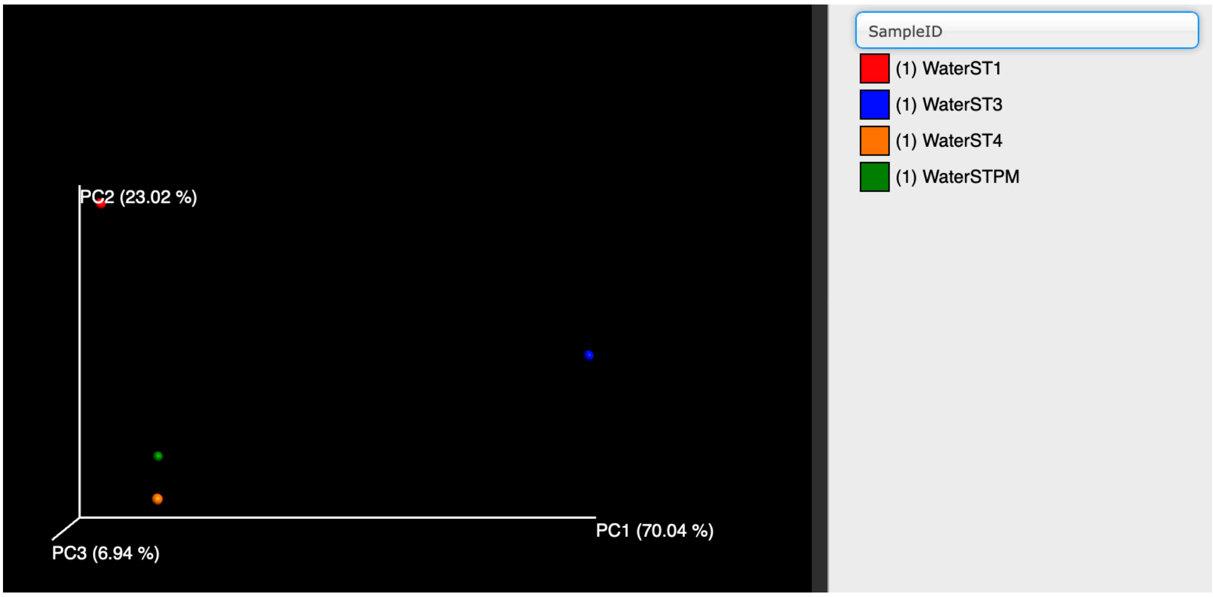

Figure S2. A PCA of weighted UniFrac distances

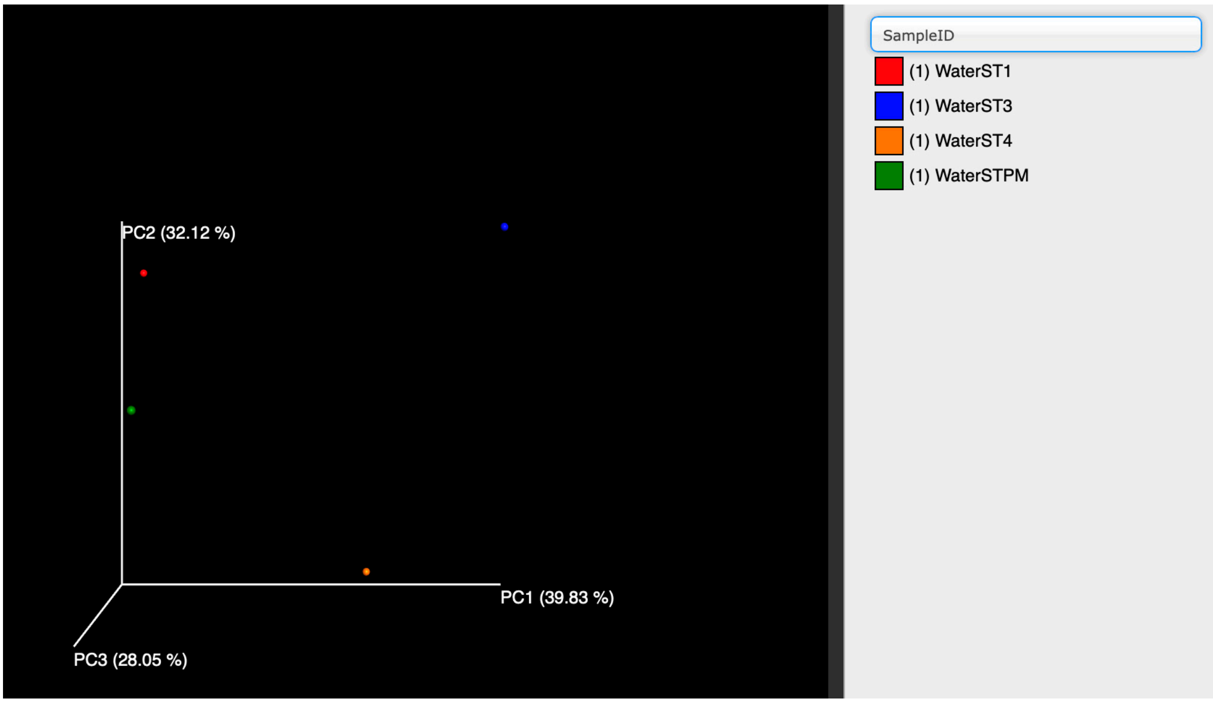

Figure S3. A PCA of unweighted UniFrac distances

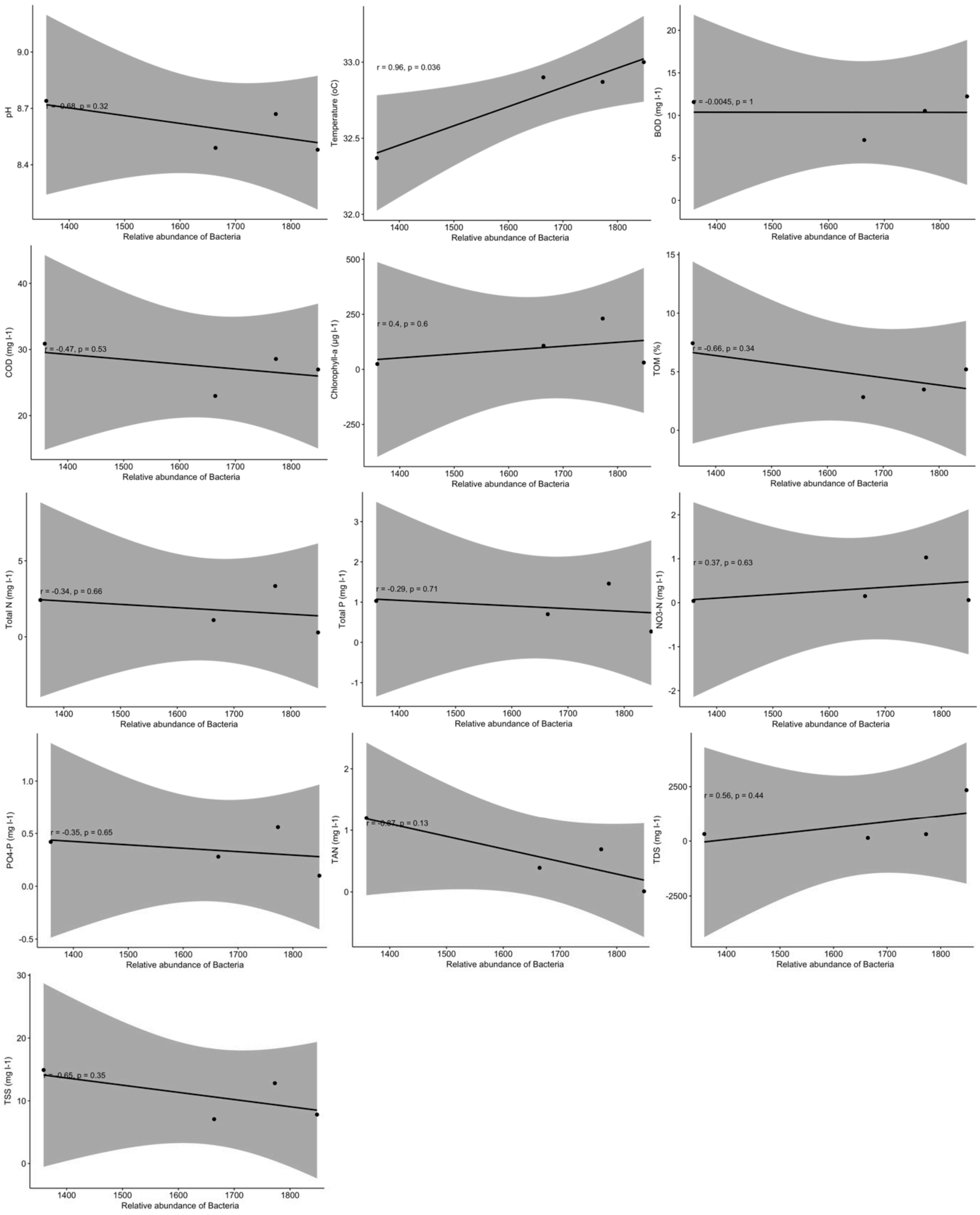

Figure S4. Pearson correlation between environmental factors and relative abundance of bacteria.

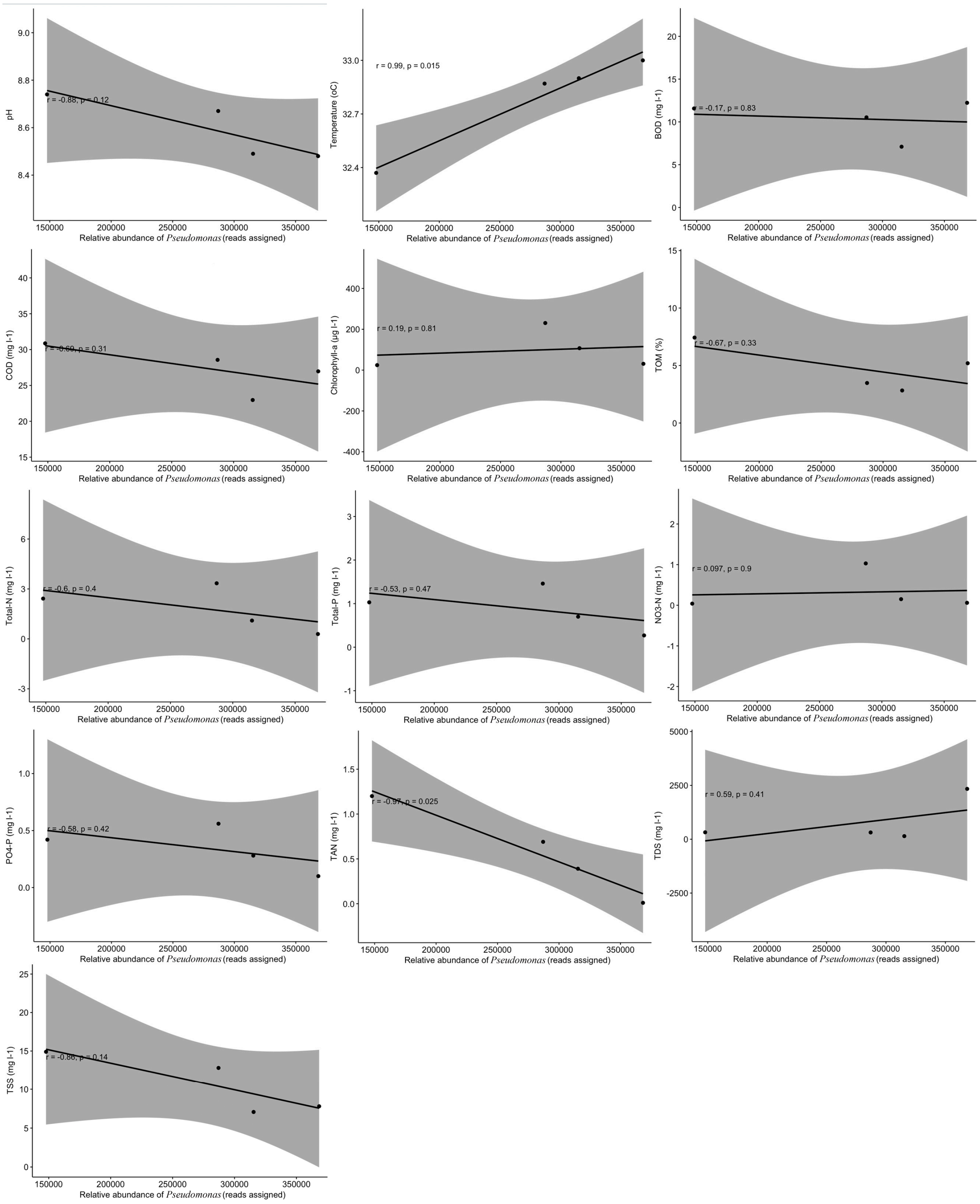

Figure S5. Pearson correlation between environmental factors and relative abundance of *Pseudomonas*.

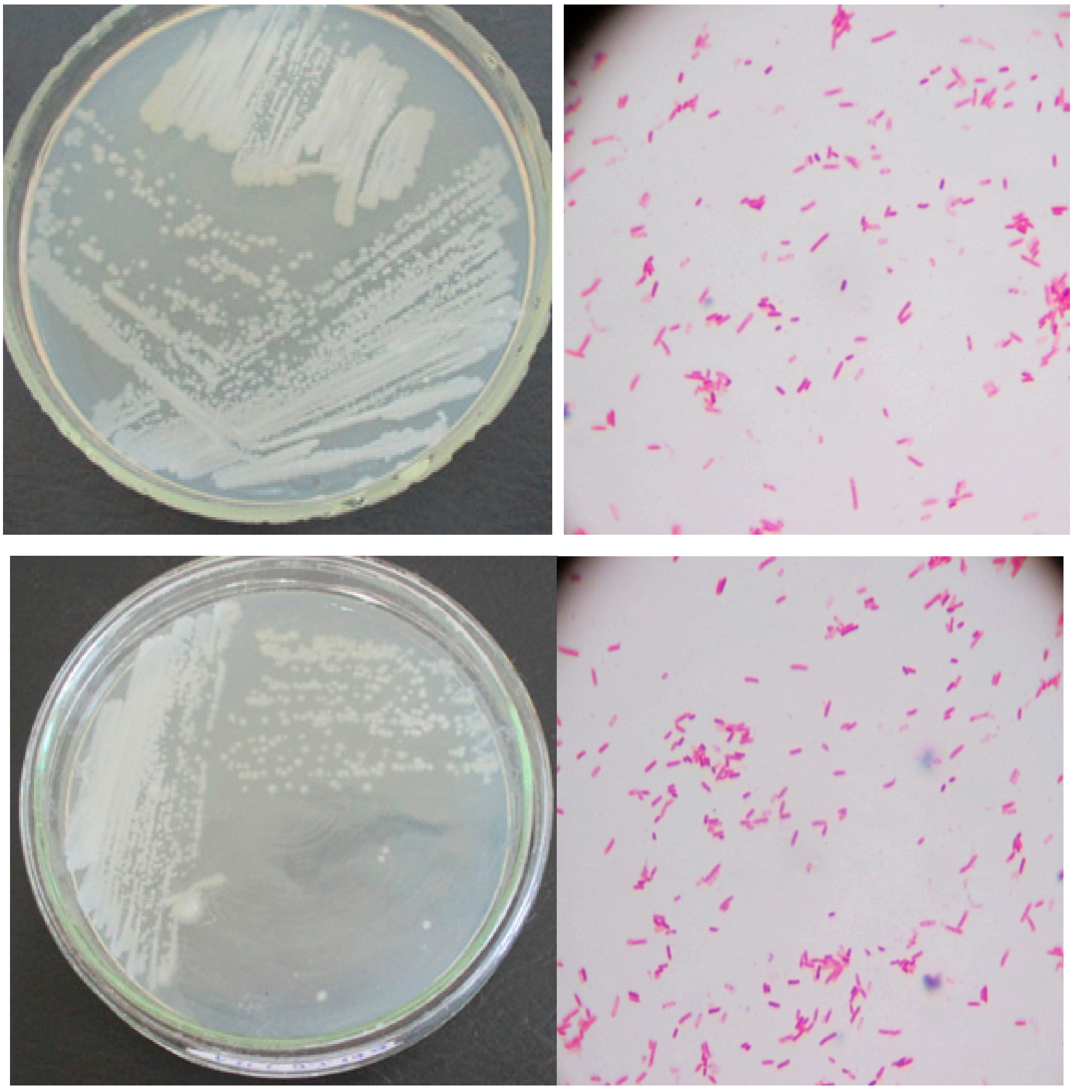

Figure S6. Gram staining test on TT322 and B12 strains
